# Establishment of a Pseudovirus-Based Golden Hamster Model for the Attachment and Entry Stages of Hendra Virus Infection and Evaluation of Protective Immunity

**DOI:** 10.3390/pathogens14090910

**Published:** 2025-09-10

**Authors:** Tao Li, Binfan Liao, Danfeng Li, Jie Zhang, Chunhui Zhao, Yunfei Pei, Liping Chen, Meng Wang, Yawen Liu, Xi Wu, Weijin Huang, Jianhui Nie

**Affiliations:** 1 Division of HIV/AIDS and Sexually-Transmitted Virus Vaccines, Institute for Biological Product Control, National Institutes for Food and Drug Control (NIFDC), Beijing 102629, China; litao@nifdc.org.cn (T.L.); zhangjie2024@nifdc.org.cn (J.Z.); zhaochunhui@nifdc.org.cn (C.Z.); 18738625256@163.com (L.C.); wangmeng@nifdc.org.cn (M.W.); yawenliu13@163.com (Y.L.); wuxi@nifdc.org.cn (X.W.); 2 State Key Laboratory of Drug Regulatory Science, Beijing 102629, China; 3 Key Laboratory of the Ministry of Health for Research on Quality and Standardization of Biotech Products, Beijing 102629, China; 4 School of Life Science and Bio-Pharmaceutics, Shenyang Pharmaceutical University, Shenyang 110016, China; lbf1761023269@163.com; 5 Division of Pharmacology, Toxicology and Biological Products Inspection, Guangxi Institutes for Food and Drug Control, Nanning 530021, China; lidanfeng0771@126.com; 6 Department of Safety, National Institutes for Food and Drug Control (NIFDC), Beijing 102629, China

**Keywords:** Hendra virus, pseudovirus, infection model, bioluminescence imaging

## Abstract

Objective: Establish an in vivo evaluation model focused on the attachment and entry stages of Hendra virus infection for protective immunity assessment. Methods: A golden hamster infection model based on recombinant Hendra-F/G pseudovirus was developed, and a luciferase luminescence assay was used to assess the optimal pseudoviral challenge in terms of route of infection, dose and detection time. The biodistribution of the pseudovirus in infected organs was evaluated using the IVIS spectral CT system. The protective effect of antibody prophylaxis was evaluated by measuring the luminescence intensity of pseudoviruses. Results: Intraperitoneal injection was identified as the optimal route of infection, and the optimal time of detection was 6 h post-challenge. Our model simulates the infection of the brain and lungs by live viruses, with the strongest infection occurring in the abdomen, especially in the intestinal organs. The dose of pseudovirus was linearly correlated with luminescence intensity. The infection model was able to differentiate the protective effect of monoclonal antibodies, with complete protection in the high-dose group. Conclusions: The recombinant Hendra-F/G pseudovirus hamster model allows the effective evaluation of prophylactic monoclonal antibodies, providing a crucial tool for studying Hendra virus infection and control strategies.

## 1. Introduction

Hendra virus (HeV), a zoonotic pathogen that emerged in the 1990s, causes severe cerebrovascular inflammation and respiratory disease with a more than 50% case fatality rate in humans [1,2,3]. It belongs to the genus Henipavirus in the Paramyxoviridae family, along with Nipah virus, with which it exhibits cross-neutralization [3,4]. The natural hosts of HeV are fruit bats, from which it is transmitted to humans across species via horses as intermediate hosts [3,5,6]. HeV has been classified as a biosafety level 4 (BSL-4) pathogen due to the high mortality rate of infected humans and the lack of treatment options [7,8,9]. In recent years, new Henipavirus members have also been identified in China, such as the Mojiang virus [10] and Langya virus [11]. Furthermore, the presence of antibodies against Nipah or closely related viruses has been confirmed in Chinese bats [12]. These findings underscore the potential risk of transmission of HeV in China and on a global scale. Significant progress has been made in the study of HeV, especially with regard to transmission routes [3], classification of the virus [8], diagnostic methods [13], and equine vaccine research [14]. However, there is still a lack of effective human vaccines and therapeutic drugs due to the high biosafety level required to handle the live virus and the associated complexity of research and clinical evaluation [2]. As a consequence, the prevention and control of HeV are still facing great challenges.

In order to better study the pathogenic mechanisms as well as the prevention and control of HeV, it is crucial to establish safe and effective animal infection models. The Syrian golden hamster is a frequently utilized experimental animal model [15] that has been employed in the study of numerous viruses, including Nipah [16,17] and SARS-CoV-2 [18]. Consequently, this study utilized a recombinant VSV pseudovirus expressing luciferase and lacking G protein, whose envelope consists of the glycoprotein (G) and fusion protein (F) proteins of Hendra virus (rVSV ΔG-HeV F/G-Fluc), to establish a pseudotyped HeV golden hamster infection model. It provides an effective tool for evaluating drugs designed to prevent the attachment and entry stages of HeV infection.

## 2. Materials and Methods

### 2.1. Cells and Plasmids

HEK293T cells (American type culture collection [atcc], crl-3216) were used for pseudovirus expression and titration in conjunction with expression plasmid pcdna3.1(+) [19]. The hek293t cells were maintained in Dulbecco’s modified eagle’s medium (dmem, high sugar; hyclone, Logan, UT, USA) supplemented with 100 µ/mL penicillin–streptomycin solution (gibco, Grand Island, NY, USA), 10% fbs (hyclone, Logan, UT, USA) and 20 mm n-2 hydroxyethylpiperazinen-2-ethanesulfonic acid (hepes, gibco, Grand Island, NY, USA) in a 5% CO_2_ environment at 37 °C and passaged every 2–3 days. The f and g genes of the Australian strain of hev (genbank No. JN255818.1) were synthesized by General Biologicals (Chuzhou, Anhui, China) Co., Ltd. with humanized codon optimization. The Kozak sequence GCCACC was added in front of the start codon, and then inserted into the pcDNA3.1(+) plasmid for transfection and expression.

### 2.2. Preparation and Titration of Pseudoviruses

HeV pseudoviruses were generated and titrated as previously described [20]. Following the instructions for the Lipofectamine 3000 transfection reagent (Invitrogen, Thermo Fisher Scientific, Carlsbad, CA, USA; catalog no. L3000015), HEK293T cells were co-transfected with vectors expressing the F and G proteins of HeV (GenBank No. JN255818.1) at a 1:2 DNA mass ratio, and an appropriate amount of recombinant Vesicular Stomatitis Virus (rVSV)-G*ΔG-Fluc virus (VSV G pseudovirus, kerafast, Boston, MA, USA) was added. The medium was replaced with fresh medium after 6 h. Thereafter, the cells were incubated at 5% CO_2_ and 37 °C for 24 h. The culture supernatant was collected and filtered through a 0.2 μm pore-size sterile filter membrane (pALL corporation, Port Washington, NY, USA; catalog no. 4612) to obtain a recombinant pseudovirus containing HeV-F/G protein (rHeV-F/G pseudovirus), which was frozen at −80 °C until further use. The 50% tissue culture infectious dose (TCID_50_) of rHeV-F/G pseudovirus was determined by titration, and the pseudovirus was diluted in 96-well plates (corning, Corning, NY, USA) in 3-fold gradients of 10, while the last column was used as a cellular control without the addition of pseudovirus. Then, 40,000 cells were added per well. After 24 h of incubation at 5% CO_2_ and 37 °C, the supernatant of the 96-well plate was discarded, and 100 μL of Fluc fluorescence detection reagent (perkinelmer, Waltham, MA, USA) was added to each well with slight shaking, after the luminescence intensity was measured in an EnSight^TM^ multimode plate reader (perkinelmer, Waltham, MA, USA). TCID_50_ values were calculated using the Reed-Muench method [21].

### 2.3. Animal Experiments

Four-week-old female Syrian golden hamsters were purchased from Beijing Viton Lihua Laboratory Animal Technology Co., Ltd. (Animal License No.: SYXK 2022-0052), and kept in the Animal Laboratory of the National Institutes for Food and Drug Control (NIFDC) after purchase. The experimental animals were randomly assigned to undergo pseudovirus challenge assays to determine the optimal infection route, detection time, and challenge dose, or to evaluate the protective effect of antibodies. Groups based on challenge routes include intracerebral (IC) injection, intraperitoneal (IP) injection, intrathoracic (IT) injection, and intranasal (IN) administration. The negative control (NC) group was not subjected to challenge. Experiments to determine the optimal detection time were conducted using four golden hamsters, two injected intraperitoneally with D-luciferin substrate (xenogen-caliper life sciences, Hopkinton, MA, USA) for luminescence imaging immediately after the injection of pseudovirus (0 h). To avoid mixing of the pseudovirus with the luminescent substrate in the intraperitoneal cavity during the 0 h assay to affect the subsequent infection with the pseudovirus, the luminescence assay at 2–96 h was conducted using 2 additional golden hamsters. The pseudovirus challenge dose for the experiments for determining the optimal challenge route and detection time was 1.0 × 10^6^ TCID_50_, and the luciferase luminescence intensity was measured using an IVIS Spectrum CT imaging system (perkinelmer, Waltham, MA, USA) 6 h after injection.

For the animal protection assay, monoclonal antibodies (mAbs) m102.3, 5B3, and 3C9 were serially diluted in phosphate-buffered saline (PBS) in a 3-fold gradient, one day prior to the challenge. The mAbs were then administered via the intramuscular route with doses of 9 mg/kg, 3 mg/kg, 1 mg/kg, 0.33 mg/kg, and 0 mg/kg according to the weight of the animals. Each dose group contained three golden hamsters. Pseudovirus challenge was performed via the IP route at a dose of 6.41 × 10^5^ TCID_50_, and the hamsters were tested for bioluminescence 6 h after challenge.

### 2.4. In Vivo Bioluminescence Imaging

Bioluminescence was imaged on an IVIS Spectrum CT system (perkinelmer, Waltham, MA, USA). Golden hamsters received an intraperitoneal injection of D-luciferin (150 μg/g), and 5 min later were anesthetized with 2.5% isoflurane (lunan pharmaceutical, Linyi, Shandong, China) prior to imaging. The bioluminescence signal of each hamster was detected with a 1 min acquisition time, and the imaging area was used to calculate the luminescence intensity in terms of total flux (p/s/cm^2^/sr). The range of the luminescence display was established from 1.5 × 10^4^ to 5 × 10^4^ p/s/cm^2^/sr. In the challenge route experiment, the width and height of the Region of Interest (ROI) in the detection system were set as follows: head (3.5 × 4.8 cm), thorax (5.5 × 2.7 cm), abdomen (6.0 × 6.8 cm), and entire body (6.8 × 16.0 cm). For other experiments, data collection utilized a full-body ROI (6.8 × 16.0 cm). Radiance data collection was performed using uniform ROI settings. Multimodal image fusion technology was used to select the bioluminescence and DLIT modules, utilizing luminescence and CT imaging technologies to perform tomographic scanning of optical signals within animals, collecting multi-channel spectra in the 560–640 nm wavelength range. Functional and structural image fusion reconstruction was performed using system software to reconstruct the localization of the bioluminescence source.

### 2.5. In Vitro Pseudovirus Neutralization Assay

As described in the Nipah virus pseudovirus neutralization assay [21,22], the extent to which rHeV-F/G pseudoviruses are neutralized was reflected by a decrease in luciferase expression. Antibodies in the serum bound to the pseudoviruses and blocked their entry into cells, resulting in a decrease in luciferase expression by the infected cells. The serum dilution corresponding to 50% inhibition of luciferase activity was defined as the half-maximal inhibitory dilution (ID_50_). A virus control (virus + cells) and a cell blank control well (cells only) were included, whereby the relative light unit (RLU) value of the cell control well was subtracted from the RLU values of all wells. The rHeV-F/G pseudovirus was pre-incubated with serum samples diluted in a 3-fold gradient in a cell culture incubator for 1 h. Then, 5 × 10^4^ 293T cells were added to each assay well. After continued culture for 48 h at 37 °C and 5% CO_2_, luciferase substrate was added as described for the pseudovirus titration assay, and RLU values were measured using a spectrophotometer. Hamster sera were tested in triplicate, and the ID_50_ values were calculated using the Reed-Muench method [21].

### 2.6. Statistical Analysis

GraphPad Prism 8.0 software (GraphPad Software Inc., San Diego, CA, USA) was used for analyzing data and graphing. Unless otherwise stated, results were expressed as the mean ± standard deviation (SD). The Reed-Muench method was employed to calculate the median effective dose, and a correlation analysis was conducted using linear regression. Differences between samples and the control group were analyzed using single-factor analysis of variance (one-way ANOVA), and differences with *p*-values of less than 0.05 were considered significant. All golden hamsters were included in the analysis.

## 3. Results

### 3.1. Assessment of Different Challenge Modes for rHeV-F/G Pseudoviruses

**Figure 1:**
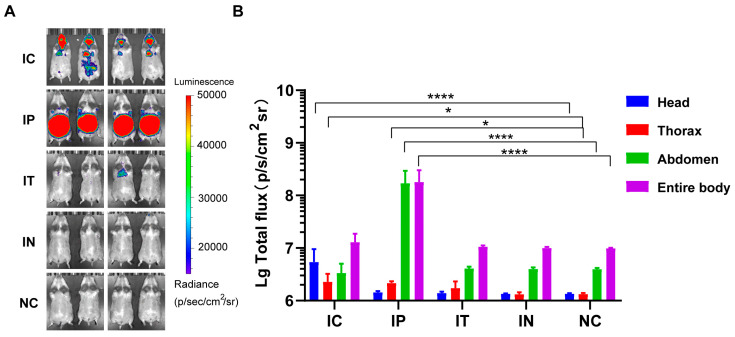
Bioluminescence in golden hamsters following rHeV-F/G pseudovirus infection via IC, IT, IP, and IN routes. (**A**) rHeV-F/G pseudovirus challenge via IC, IT, IP, and IN routes, followed by injection of D-luc luminescent substrate at 6 h post-challenge. Luciferase bioluminescence was detected and imaged utilizing the IVIS Spectrum CT imaging system, *n* = 4 hamsters per group. The luminescence intensity label displayed ranges from 1.5 × 10^4^ to 5 × 10^4^ p/s/cm^2^/sr. (**B**) Different ROIs were set for the head, thorax, abdomen, and entire body, respectively, and radiance signals were collected using the IVIS Spectrum CT system. Statistical analyses were performed on the log-transformed values of the total fluxes for the different challenge routes, with four independent biological replicates of the data in each group, and compared with the NC group separately. One-way ANOVA was applied. Data are expressed as the mean ± SD. * *p* < 0.05, **** *p* < 0.001.

To identify effective routes for assessing rHeV-F/G pseudovirus infection, we selected IC, IT, IP, and IN infection, which are frequently used in animal models. As shown in Figure 1, the IC group exhibited luminescence in the brain, and lC injection of the pseudovirus resulted in infection of the thoracic cavity, which was consistent with the fact that authentic live HeV can trigger encephalitis and respiratory symptoms. Comparisons of the IT and IN groups with the NC group showed that only one hamster in each group exhibited weak luminescence in the oral cavity and thoracic cavity, without significant differences in luminescence intensity (Figure 1B). Notably, the IP group exhibited strong luminescence in all four golden hamsters, with a more homogeneous degree of infection. The statistical analysis of the total luminescence intensity revealed a significant difference between the IP and NC groups (*p* < 0.05). Analysis of luminescence intensity collected from the entire body revealed that the IP group exhibited a higher infection intensity than the IC group, as illustrated in Figure 1B. The aforementioned results indicate that the IP route for rHeV-F/G pseudovirus infection is a superior challenge route.

### 3.2. Assessment of the Optimal Detection Time for the rHeV-F/G Pseudovirus Infection Model

**Figure 2:**
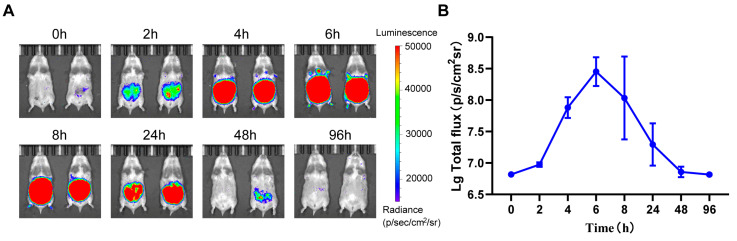
Bioluminescence intensity over time after rHeV-F/G pseudovirus challenge. (**A**) Challenge was performed via the IP route, followed by luciferase bioluminescence imaging analysis at 0, 2, 4, 6, 8, 24, 48, and 96 h post-challenge, *n* = 2 hamsters per group. The luminescence intensity label displayed ranges from 1.5 × 10^4^ to 5 × 10^4^ p/s/cm^2^/sr. (**B**) Log-transformed values of luminescence detected at different time points are shown after injection of rHeV-F/G pseudovirus via the IP route. The values corresponding to each time point are 2 independent replicates of the biological samples, and the data are expressed as the mean ± SD.

After identifying intraperitoneal injection as the optimal challenge route for rHeV-F/G pseudovirus in golden hamsters, the optimal luminescence detection time following pseudovirus challenge was further assessed. The fixed challenge dose of 1.0 × 10^6^ TCID_50_ was utilized, and the results of the post-challenge interval testing are displayed in Figure 2A. The intensity of bioluminescence exhibited a gradual increase over time following the challenge. The maximal luciferase bioluminescence intensity was detected at 6 h post-challenge, with an average luminescence intensity of 3.03 × 10^8^ (p/s/cm^2^/sr). Thereafter, the luminescence gradually decreased until 96 h, at which point it was essentially undetectable (Figure 2B). In light of the aforementioned results, it was determined that the optimal luminescence detection time for the evaluation of rHeV-F/G pseudovirus infection in the golden hamster model was 6 h after challenge.

### 3.3. Biodistribution Characterization of rHeV-F/G Pseudovirus-Infected Golden Hamsters

**Figure 3:**
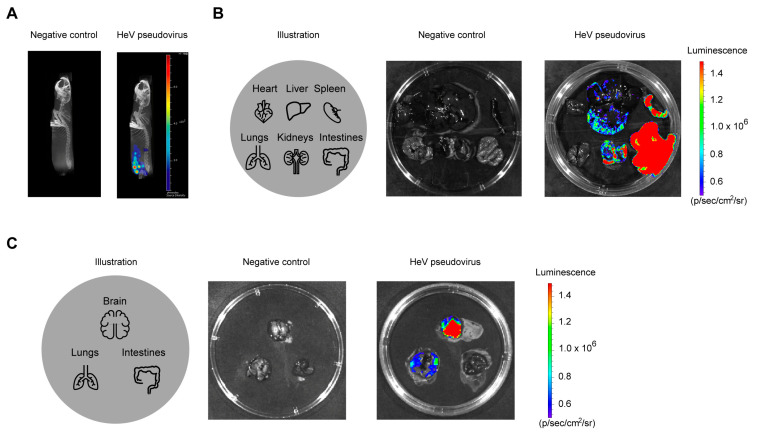
In vivo biodistribution analysis after rHeV-F/G pseudovirus challenge. (**A**) Golden hamsters challenged with rHeV-F/G pseudovirus via the IP route were subjected to CT bioluminescence imaging to demonstrate the distribution of luciferase luminescence in vivo 6 h after infection. The negative control hamster was not challenged but only injected with luminescent substrate and imaged in the same way. The luminescence intensity label displayed ranges from 1.6 × 10^5^ to 7.8 × 10^5^ photons/s. (**B**) The left panel illustrates the arranged positions of the heart, liver, spleen, lungs, kidneys, and intestines, the middle panel shows the bioluminescence of the abdominal organs of a golden hamster in the NC group, and the right panel shows the bioluminescence of the abdominal organs of a rHeV-F/G pseudovirus-challenged golden hamster. The luminescence intensity label displayed ranges from 5 × 10^5^ to 1.5 × 10^6^ p/s/cm^2^/sr. (**C**) The left panel illustrates the arranged positions of the brain, lungs and intestines, the middle panel shows the bioluminescence of the abdominal organs of a golden hamster in the NC group, and the right panel shows the bioluminescence of the abdominal organs of a rHeV-F/G pseudovirus-challenged golden hamster. The luminescence intensity label displayed ranges from 5 × 10^5^ to 1.5 × 10^6^ p/s/cm^2^/sr.

As indicated in this study, the optimal challenge route was determined to be intraperitoneal injection of 1.0 × 10^6^ TCID_50_ of rHeV-F/G pseudovirus, which was then allowed to infect tissues for a period of 6 h. The biodistribution of the pseudovirus-infected sites was subsequently demonstrated by CT imaging, as illustrated in Figure 3A. Compared with the NC group, luminescence in hamsters infected with rHeV-F/G pseudovirus in the abdominal cavity was mainly concentrated in the lower abdomen. The control and challenged golden hamsters were subjected to further dissection, after which the heart, liver, spleen, lungs, kidneys, and intestines were examined for bioluminescence. The results indicated that the intestines exhibited the highest luminescence intensity, followed by the spleen, kidneys, and liver (Figure 3B). In addition, we also dissected the hamsters that underwent IC challenge and confirmed that this route induced bioluminescence in the brain and lungs (Figure 3C). The above results suggest that rHeV-F/G pseudoviruses can infect the brain, lungs, and abdominal organs, with especially intense infection in the intestinal organs.

### 3.4. Dose Optimization for rHeV-F/G Pseudovirus Infection

**Figure 4:**
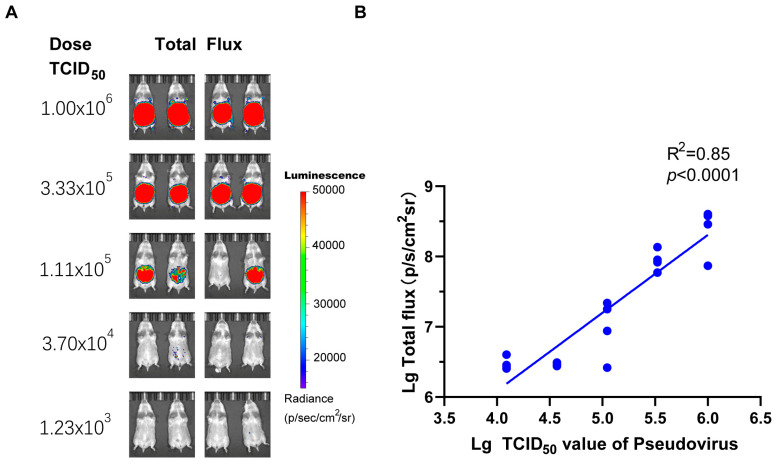
Correlation of bioluminescence intensity with the rHeV-F/G pseudovirus challenge dose. (**A**) Pseudovirus challenge was performed by intraperitoneal injection at a 3-fold gradient dilution with a maximum starting dose of 1.0 × 10^6^ TCID_50_, *n* = 4 hamsters per group. The luminescence intensity label displayed ranges from 1.5 × 10^4^ to 5 × 10^4^ p/s/cm^2^/sr. (**B**) The log-transformed values of the TCID_50_ values of the injected pseudovirus dose and the log-transformed values of the total luminescence intensity of each group were subjected to a linear regression analysis, and a robust correlation was demonstrated (R^2^ = 0.85, *p* < 0.001).

The objective of this analysis was to ascertain the optimal dosage of rHeV-F/G pseudovirus for in vivo infection in golden hamsters. To this end, a series of five gradients was established, starting at 1.0 × 10^6^ TCID_50_ with subsequent 3-fold gradient dilution. As shown in Figure 4, the log-transformed values of the challenge dose of the pseudovirus and the log-transformed value of the luciferase luminescence intensity of the golden hamsters exhibited a robust linear correlation (R^2^ = 0.85, *p* < 0.0001). Using the Reed-Muench method, the median animal infectious dose (AID_50_) was determined to be 6.41 × 10^4^ TCID_50_. When the challenge doses were 1.0 × 10^6^ and 3.33 × 10^5^ TCID_50_, the infection of all golden hamsters could be achieved, and the total flux intensity of the infection in the two dose groups was stable (Figure 4A), with a relative standard deviation (RSD) of the logarithmic value of the total flux intensity being less than 5%. Consequently, a pseudoviral challenge of approximately 10 times the AID_50_ was found to be suitable for subsequent evaluation of the protective effect of a monoclonal antibody (mAb) or vaccine.

### 3.5. Evaluation of Protection Using the rHeV-F/G Pseudovirus Golden Hamster Infection Model

**Figure 5:**
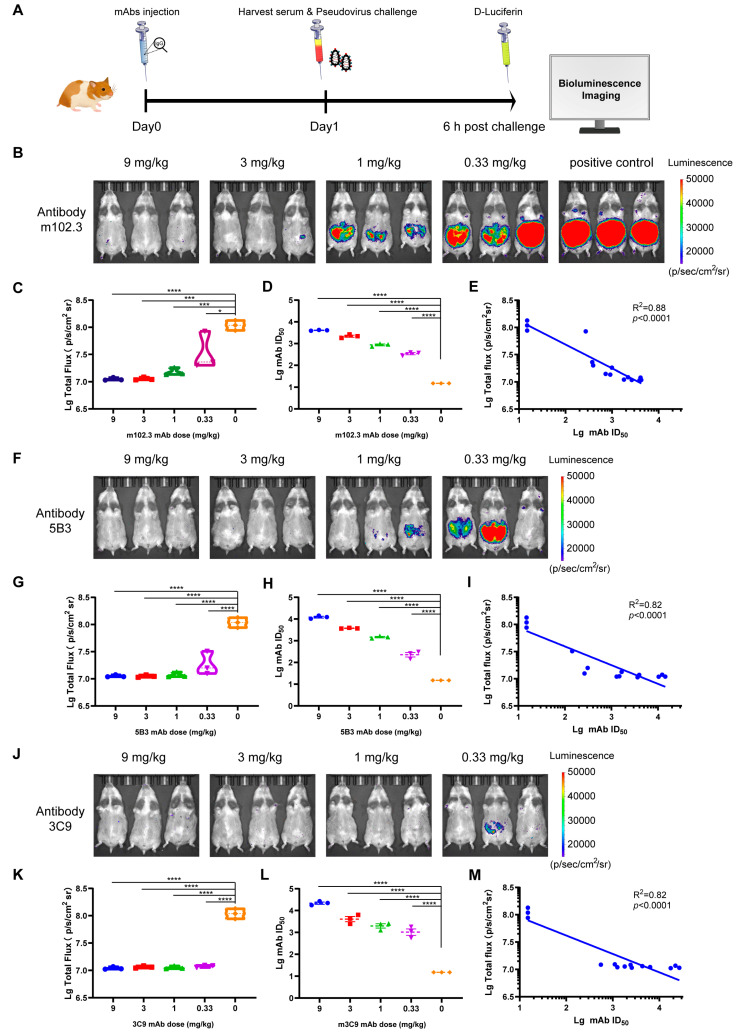
Evaluation of mAb protection using the rHeV-F/G pseudovirus golden hamster infection model. (**A**) The efficacy of antibodies in preventing HeV infection was evaluated using a 3-fold antibody dose gradient, with a maximum dose of 9 mg/kg. Each group consisted of 3 golden hamsters, which were intramuscularly injected with different doses of antibodies 1 day prior to challenge. Serum was collected prior to challenge to measure antibody titers, and bioluminescence was detected and imaged at 6 h post-challenge. (**B**,**F**,**J**) Total luminescence values of golden hamster models with different antibody titers following rHeV-F/G pseudovirus infection. The data represent three independent biological replicates. In accordance with the 3R principles for animal experimentation and to ensure standardized inter-antibody comparisons, a single cohort of infected positive-control animals was employed. The luminescence intensity label displayed ranges from 1.5 × 10^4^ to 5 × 10^4^ p/s/cm^2^/sr. (**C**,**G**,**K**) The logarithmic values of total luminescence values and serum antibody titers for each experimental group were compared with the control group (antibody dose of 0 mg/kg) using one-way ANOVA. (**D**,**H**,**L**) Serum titer data were measured in triplicate, and results are expressed as the mean ± standard error of the mean (SEM). * *p* < 0.05, and *** *p* < 0.005, **** *p* < 0.001. (**E**,**I**,**M**) Linear regression analysis of the logarithmic values of antibody concentrations and total luminescence intensities for each group of injection doses showed good linear relationships, with R^2^ values > 0.8 and *p* < 0.001.

The objective of this experiment is to utilize the rHeV-F/G pseudovirus golden hamster infection model to evaluate the potential in vivo protective efficacy of the anti-NiV-G mAb m102.3 [23], as well as the anti-NiV-F mAbs 5B3 [1] and 3C9 [20], which are highly effective at neutralizing HeV in vitro. For this purpose, antibodies were administered via intramuscular injection one day prior to the challenge, with an initial dose of 9 mg/kg (Figure 5A). The mAbs were then serially diluted 3-fold, resulting in four dilution gradients. On the first day following the antibody injection, serum was collected from the golden hamsters for antibody titer detection using the rHeV-F/G pseudovirus. The protective efficacy of the antibodies was assessed 6 h post-infection. In the mAb m102.3 protection experiment, the high-dose group (9 mg/kg) achieved complete protection against abdominal infection by rHeV-F/G pseudovirus (Figure 5B). As the mAb dose was gradually reduced, the total fluorescence intensity produced by pseudovirus infection increased progressively until the dose was reduced below 1 mg/kg, at which point it was no longer effective in preventing HeV infection in golden hamsters (Figure 5B,C). In vivo, the median effective dose (AED_50_) of mAb m102.3 in animals was 2.25 mg/kg. It was observed that when the titer of the m102.3 mAb in serum reached 2196 or above, the golden hamsters were completely protected against abdominal infection (Figure 5D). In vitro neutralization assays demonstrated that the log ID_50_ value of the neutralizing antibody titer in the serum obtained prior to challenge was inversely proportional to the log-transformed total flux intensity (Figure 5E).

In addition to evaluating the in vivo protective efficacy of the anti-HeV-G mAb m102.3, we also evaluated the anti-HeV-F mAbs 5B3 and 3C9. As shown in Figure 5F, the protective efficacy of 5B3 was higher than that of m102.3, and it could prevent rHeV-F/G pseudovirus infection at doses of 9 and 3 mg/kg (Figure 5F,G). A similar dose-dependent protective effect was observed for the 5B3 mAb in the HeV hamster infection model. The median animal-effective dose (AED_50_) of the 5B3 mAb was 1.29 mg/kg, with a serum mAb titer of 1652, achieving complete protection against HeV (Figure 5H,I). The 3C9 mAb achieved the best in vivo protective effect in the hamster infection model, with serum neutralization titers above 1105 providing complete protection against rHeV-F/G pseudovirus challenge (Figure 5J–M). Even at a dose of 0.33 mg/kg, only one hamster showed mild luminescence post-challenge (Figure 5J). This in vivo experimental result was consistent with the previous results of in vitro neutralization efficacy assessment [20]. The findings from in vivo protection experiments demonstrated a substantial dose-dependent effect of the mAb on the total luminescence intensity in pseudovirus-challenged golden hamsters. These results suggest that the golden hamster rHeV-F/G pseudoviral infection model can be used to assess and quantify the efficacy of mAbs in inhibiting infection in vivo.

## 4. Discussion

This study successfully established a golden hamster model with rHeV-F/G pseudovirus for evaluating HeV attachment- and entry-targeting prophylactics in vivo. The optimum of infection route, detection time, and challenge doses in vivo bioluminescence in golden hamsters were evaluated using the rHeV-F/G pseudovirus packaged using the VSV backbone system containing the Fluc reporter gene. In this study, rHeV-F/G pseudoviruses produced luminescence in the thoracic and cephalic of golden hamsters by IC route. However, the challenges of the IP route produced the most pronounced infection, achieving consistent positive infection within the group. The present study found that the IP route is the optimal infection pathway in the rHeV-F/G pseudovirus model, consistent with the live HeV infection route in a hamster model [15]. Lethality could be achieved at lower doses by IP challenge in an infection with a live NiV, which is in the same genus as HeV, and IP-infected animals also had a shorter time to death than hamsters administered IN [16]. Another study also found that IP challenge caused the virus to spread through the tissue more quickly and that survival was homogeneous for the same challenge dose [24]. The biodistribution of the pseudovirus in vivo was monitored as well. The results demonstrated that the rHeV-F/G pseudovirus was capable of infecting multiple organs in golden hamsters, such as the brain, lungs, and intestines, which was consistent with the authentic Henipavirus infection [25,26]. In another study employing a mouse infection model with rHeV-F/G pseudovirus, the same VSV pseudovirus system was utilized. This system was capable of inducing infection-related luminescence in the thorax of mice [20]. The observed discrepancy in infection rates may be ascribed to the characteristic tissue tropism of the virus, which may differ among species. In the present study, rHeV-F/G pseudovirus exhibited robust infection-induced luminescence, predominantly in the intestinal tract. Notably, intestinal epithelial cells [27] and stem cells [28] were previously found to express the HeV receptor protein Ephrin-B2. Consequently, this may render the intestinal tract of golden hamsters susceptible to rHeV-F/G pseudovirus infection.

Furthermore, evaluation of the protective capacity of antibodies using the rHeV-F/G pseudovirus golden hamster infection model was substantiated by employing the mAbs m102.3 and 5B3, which have been evaluated for their neutralizing capacity against the live virus at the cellular level [1,23]. A negative correlation between the dose of monoclonal antibodies and the luminescence intensity in the golden hamster infection model was effectively demonstrated. In addition, the results of the in vivo protective efficacy of antibodies assessed using the golden hamster infection model were consistent with the neutralizing activity previously detected at the cellular level [20]. The 3C9 mAb had the highest protective efficacy and provided protection in almost all antibody gradients, followed by 5B3 and m102.3. In this study, mAbs m102.3 and 5B3 achieved complete infection inhibition at a high dose of 9 mg/kg, with AED_50_ values of 2.25 and 1.29 mg/kg, respectively. By contrast, a dose of 20 mg/kg of 5B3 humanized antibody resulted in the survival of all treated ferrets in a previous live-virus study, but the authors did not determine the efficacy at lower doses [29]. In a live virus challenge experiment with ferrets, administration of a 50 mg dose of m102.4 (a mAb derived from m102.3) 10 h after a lethal NiV challenge completely protected the ferrets from disease [30]. In another live-virus study, Nip GIP21 mAb rescued more than half of the hamsters at a dose of 3.0 mg/kg [15]. Most of these live virus studies used a fixed dose for evaluation. Here, the use of a pseudovirus infection model in golden hamsters allowed a more objective quantification of protective efficacy. The successful establishment of this model provides a safe and effective platform for HeV research, helping to address the biosafety risks while also reducing the difficulty of quantifying the protective efficacy of antibodies.

In addition to evaluating the prophylactic effect of antibodies using the rHeV-F/G pseudovirus animal infection model in the present study, the application of pseudoviruses allows for the visual and quantitative evaluation of vaccine protective efficacy in animal models. This has already been applied in vaccine studies for human papillomavirus, SARS-CoV-2 virus, and EV 71 enterovirus [31,32]. Furthermore, the pseudovirus technique has been employed to assess the efficacy of pharmaceutical agents designed to impede viral invasion, thereby highlighting the potential for antibody-based drugs to fulfill a significant role in the therapeutic management of viral infections [17,33]. These findings highlight the potential to extend the golden hamster infection model to a wider range of applications. Notwithstanding the advantages of the pseudoviral golden hamster infection model in Hendra virus studies, such as safety, ease of operation, and intuitive quantification, there are undeniable limitations. For instance, it is not feasible to fully recapitulate the infection process of live viruses, and the transient single-round infection of pseudoviruses may not accurately mimic the resulting pathological lesions. This infection model has been demonstrated to be a valuable tool in the screening of prophylactic antibody drugs. However, since only the HeV-F/G proteins are recombinant into the pseudovirus and not all HeV proteins are included, the final candidate drug evaluation should still undergo challenge validation with live HeV.

## 5. Conclusions

In summary, this study provides a safer, more convenient and intuitively quantifiable alternative method by establishing a rHeV-F/G pseudovirus golden hamster infection model, which provides an important experimental basis for the in-depth study of the evaluation of drug effectiveness.

## Data Availability

Data supporting the findings of this study are available from the corresponding authors upon reasonable request.

## References

[1] Dang H.V., Chan Y.P., Park Y.J., Snijder J., Da Silva S.C., Vu B., Yan L., Feng Y.R., Rockx B., Geisbert T.W. (2019). An antibody against the F glycoprotein inhibits Nipah and Hendra virus infections. Nat. Struct. Mol. Biol..

[2] Pigeaud D.D., Geisbert T.W., Woolsey C. (2023). Animal Models for Henipavirus Research. Viruses.

[3] Murray K., Selleck P., Hooper P., Hyatt A., Gould A., Gleeson L., Westbury H., Hiley L., Selvey L., Rodwell B. (1995). A morbillivirus that caused fatal disease in horses and humans. Science.

[4] Selvey L.A., Wells R.M., McCormack J.G., Ansford A.J., Murray K., Rogers R.J., Lavercombe P.S., Selleck P., Sheridan J.W. (1995). Infection of humans and horses by a newly described morbillivirus. Med. J. Aust..

[5] Murray K., Rogers R., Selvey L., Selleck P., Hyatt A., Gould A., Gleeson L., Hooper P., Westbury H. (1995). A novel morbillivirus pneumonia of horses and its transmission to humans. Emerg. Infect. Dis..

[6] Playford E.G., McCall B., Smith G., Slinko V., Allen G., Smith I., Moore F., Taylor C., Kung Y.H., Field H. (2010). Human Hendra virus encephalitis associated with equine outbreak, Australia, 2008. Emerg. Infect. Dis..

[7] Field H., Schaaf K., Kung N., Simon C., Waltisbuhl D., Hobert H., Moore F., Middleton D., Crook A., Smith G. (2010). Hendra virus outbreak with novel clinical features, Australia. Emerg. Infect. Dis..

[8] Taylor J., Thompson K., Annand E.J., Massey P.D., Bennett J., Eden J.-S., Horsburgh B.A., Hodgson E., Wood K., Kerr J. (2022). Novel variant Hendra virus genotype 2 infection in a horse in the greater Newcastle region, New South Wales, Australia. One Health.

[9] Sharma N., Jamwal V.L., Nagial S., Ranjan M., Rath D., Gandhi S.G. (2024). Current status of diagnostic assays for emerging zoonotic viruses: Nipah and Hendra. Expert Rev. Mol. Diagn..

[10] Wu Z., Yang L., Yang F., Ren X., Jiang J., Dong J., Sun L., Zhu Y., Zhou H., Jin Q. (2014). Novel Henipa-like virus, Mojiang Paramyxovirus, in rats, China, 2012. Emerg. Infect. Dis..

[11] Zhang X.-A., Li H., Jiang F.-C., Zhu F., Zhang Y.-F., Chen J.-J., Tan C.-W., Anderson D.E., Fan H., Dong L.-Y. (2022). A Zoonotic Henipavirus in Febrile Patients in China. N. Engl. J. Med..

[12] Li Y., Wang J., Hickey A.C., Zhang Y., Li Y., Wu Y., Zhang H., Yuan J., Han Z., McEachern J. (2008). Antibodies to Nipah or Nipah-like viruses in bats, China. Emerg. Infect. Dis..

[13] Wang J., Anderson D.E., Halpin K., Hong X., Chen H., Walker S., Valdeter S., van der Heide B., Neave M.J., Bingham J. (2021). A new Hendra virus genotype found in Australian flying foxes. Virol. J..

[14] Li Y., Li R., Wang M., Liu Y., Yin Y., Zai X., Song X., Chen Y., Xu J., Chen W. (2020). Fc-Based Recombinant Henipavirus Vaccines Elicit Broad Neutralizing Antibody Responses in Mice. Viruses.

[15] Guillaume V., Wong K.T., Looi R.Y., Georges-Courbot M.C., Barrot L., Buckland R., Wild T.F., Horvat B. (2009). Acute Hendra virus infection: Analysis of the pathogenesis and passive antibody protection in the hamster model. Virology.

[16] Wong K.T., Grosjean I., Brisson C., Blanquier B., Fevre-Montange M., Bernard A., Loth P., Georges-Courbot M.-C., Chevallier M., Akaoka H. (2003). A Golden Hamster Model for Human Acute Nipah Virus Infection. Am. J. Pathol..

[17] Guillaume V., Contamin H., Loth P., Grosjean I., Courbot M.C., Deubel V., Buckland R., Wild T.F. (2006). Antibody prophylaxis and therapy against Nipah virus infection in hamsters. J. Virol..

[18] Sia S.F., Yan L.-M., Chin A.W.H., Fung K., Choy K.-T., Wong A.Y.L., Kaewpreedee P., Perera R.A.P.M., Poon L.L.M., Nicholls J.M. (2020). Pathogenesis and transmission of SARS-CoV-2 in golden hamsters. Nature.

[19] Li T., Cui Z., Jia Y., Liang Z., Nie J., Zhang L., Wang M., Li Q., Wu J., Xu N. (2022). Aggregation of high-frequency RBD mutations of SARS-CoV-2 with three VOCs did not cause significant antigenic drift. J. Med. Virol..

[20] Li T., Xu H., Zhang M., Nie J., Liao B., Xie J., Jiang Y., Liu Y., Ge P., Zhao C. (2025). A monoclonal antibody targeting conserved regions of pre-fusion protein cross-neutralizes Nipah and Hendra virus variants. Antivir. Res..

[21] Nie J., Liu L., Wang Q., Chen R., Ning T., Liu Q., Huang W., Wang Y. (2019). Nipah pseudovirus system enables evaluation of vaccines in vitro and in vivo using non-BSL-4 facilities. Emerg. Microbes Infect..

[22] Gao Z., Li T., Han J., Feng S., Li L., Jiang Y., Xu Z., Hao P., Chen J., Hao J. (2022). Assessment of the immunogenicity and protection of a Nipah virus soluble G vaccine candidate in mice and pigs. Front. Microbiol..

[23] Xu K., Rockx B., Xie Y., DeBuysscher B.L., Fusco D.L., Zhu Z., Chan Y.P., Xu Y., Luu T., Cer R.Z. (2013). Crystal structure of the Hendra virus attachment G glycoprotein bound to a potent cross-reactive neutralizing human monoclonal antibody. PLoS Pathog..

[24] Findlay-Wilson S., Flett L., Salguero F.J., Ruedas-Torres I., Fotheringham S., Easterbrook L., Graham V., Dowall S. (2023). Establishment of a Nipah Virus Disease Model in Hamsters, including a Comparison of Intranasal and Intraperitoneal Routes of Challenge. Pathogens.

[25] Singh R.K., Dhama K., Chakraborty S., Tiwari R., Natesan S., Khandia R., Munjal A., Vora K.S., Latheef S.K., Karthik K. (2019). Nipah virus: Epidemiology, pathology, immunobiology and advances in diagnosis, vaccine designing and control strategies—A comprehensive review. Vet. Q..

[26] Ang L.T., Nguyen A.T., Liu K.J., Chen A., Xiong X., Curtis M., Martin R.M., Raftry B.C., Ng C.Y., Vogel U. (2022). Generating human artery and vein cells from pluripotent stem cells highlights the arterial tropism of Nipah and Hendra viruses. Cell.

[27] Hafner C., Meyer S., Langmann T., Schmitz G., Bataille F., Hagen I., Becker B., Roesch A., Rogler G., Landthaler M. (2005). Ephrin-B2 is differentially expressed in the intestinal epithelium in Crohn’s disease and contributes to accelerated epithelial wound healing in vitro. World J. Gastroenterol..

[28] Genander M. (2012). Eph and ephrins in epithelial stem cell niches and cancer. Cell Adhes. Migr..

[29] Mire C.E., Chan Y.-P., Borisevich V., Cross R.W., Yan L., Agans K.N., Dang H.V., Veesler D., Fenton K.A., Geisbert T.W. (2020). A Cross-Reactive Humanized Monoclonal Antibody Targeting Fusion Glycoprotein Function Protects Ferrets Against Lethal Nipah Virus and Hendra Virus Infection. J. Infect. Dis..

[30] Bossart K.N., Zhu Z., Middleton D., Klippel J., Crameri G., Bingham J., McEachern J.A., Green D., Hancock T.J., Chan Y.P. (2009). A neutralizing human monoclonal antibody protects against lethal disease in a new ferret model of acute nipah virus infection. PLoS Pathog..

[31] Guillaume V., Contamin H., Loth P., Georges-Courbot M.C., Lefeuvre A., Marianneau P., Chua K.B., Lam S.K., Buckland R., Deubel V. (2004). Nipah virus: Vaccination and passive protection studies in a hamster model. J. Virol..

[32] Wang Y., Zhou Z., Wu X., Li T., Wu J., Cai M., Nie J., Wang W., Cui Z. (2023). Pseudotyped Viruses. Adv. Exp. Med. Biol..

[33] Cao Z., Jin H., Wong G., Zhang Y., Jiao C., Feng N., Wu F., Xu S., Chi H., Zhao Y. (2021). The Application of a Safe Neutralization Assay for Ebola Virus Using Lentivirus-Based Pseudotyped Virus. Virol. Sin..

